# The reliability of assigning individuals to cognitive states using the Mini Mental-State Examination: a population-based prospective cohort study

**DOI:** 10.1186/1471-2288-11-127

**Published:** 2011-09-06

**Authors:** Riccardo E Marioni, Mark Chatfield, Carol Brayne, Fiona E Matthews

**Affiliations:** 1 Department of Public Health and Primary Care, University of Cambridge, Cambridge CB2 0SR, UK; 2 Medical Research Council Biostatistics Unit, Cambridge CB2 0SR, UK

**Keywords:** MMSE, reliability, test-retest, ageing, elderly

## Abstract

**Background:**

Previous investigations of test re-test reliability of the Mini-Mental State Examination (MMSE) have used correlations and statistics such as Cronbach's α to assess consistency. In practice, the MMSE is usually used to group individuals into cognitive states. The reliability of this grouping (state based approach) has not been fully explored.

**Methods:**

MMSE data were collected on a subset of 2,275 older participants (≥ 65 years) from the population-based Medical Research Council Cognitive Function and Ageing Study. Two measurements taken approximately two months apart were used to investigate three state-based categorisations. Descriptive statistics were used to determine how many people remained in the same cognitive group or went up or down groups. Weighted logistic regression was used to identify predictive characteristics of those who moved group.

**Results:**

The proportion of people who remained in the same MMSE group at screen and follow-up assessment ranged from 58% to 78%. The proportion of individuals who went up one or more groups was roughly equal to the proportion that went down one or more groups; most of the change occurred when measurements were close to the cut-points. There was no consistently significant predictor for changing cognitive group.

**Conclusion:**

A state-based approach to analysing the reliability of the MMSE provided similar results to correlation analyses. State-based models of cognitive change or individual trajectory models using raw scores need multiple waves to help overcome natural variation in MMSE scores and to help identify true cognitive change.

## Background

The Mini-Mental State Examination (MMSE) was developed in 1975 as a brief tool to measure global cognitive function [1]. It contains nineteen items on orientation, registration, attention and calculation, recall, language, and praxis, and is scored from 0 to 30. It is primarily used as a screening test for dementia with scores below 24 commonly used to indicate a cognitive deficit. A 1998 review of the MMSE noted that it has a ceiling effect in young healthy adults and a floor effect in older, severely impaired adults [2]; ceiling and floor effects of the have also been discussed in detail elsewhere [3-5]. It has also been shown that MMSE scores are affected by age and education [3].

Despite its intrinsic limitations for measuring subtle change in ability, the MMSE is frequently used to measure cognitive change over time. Several studies have measured change as the difference in two scores [6,7] whereas others have used data from multiple waves [8,9]. When monitoring cognitive test scores over time it is desirable to account for natural variation from measurement error and test re-test reliability. Test re-test reliability of the MMSE has been investigated to a limited extent despite being of potential importance in the application of cut-points to categorise individuals for many purposes such as eligibility for medication or care support. Grouping of the MMSE variable is used in policy with dementia treatment being given to selected subgroups [10]. However, if an individual is assigned to a treatment group based on a single MMSE measure, it is vital to know how reliable such a measure is. This also applies to clinical research where MMSE cut-points are commonly used to select or reject individuals from a study or treatment regimen.

A review paper of studies analysing MMSE test-retest reliability described moderate to high correlations between measures [5]. However, it is debatable whether these are the most appropriate assessments of agreement. Correlations will measure association but not necessarily agreement [11]. Similarly, reliability as measured by Cronbach's α also relies on the calculation of intercorrelations between the two or more measures being analysed. For example, if everyone in a cohort had a one point increase in MMSE score between baseline and follow-up then the correlation between the two measures would be 1. This would imply association but not agreement. In an approach using MMSE groupings, if all individuals again scored an additional MMSE point between waves, many would remain in the same MMSE group giving better scope to measure agreement.

A statistical issue to consider when using the MMSE as a screening tool for further assessment of a sub-group of participants is regression to the mean. This phenomenon occurs when there is imperfect correlation between two measures [11]. For example, in a test re-test situation where scores at both testing occasions have the same mean and variance, the group of individuals attaining a particular score at baseline will be expected to average a score that is closer to the mean at re-test. This may account for much of the apparent cognitive decline in people with high initial scores on the MMSE.

Whilst many studies split MMSE scores into groups before analysing, the short term reliability of these groupings and the potential for misclassification has not been studied in detail. The aim of this study was to investigate the reliability of a single measure of MMSE group, as used in clinical practice, by investigating the reliability of two measures taken a short time apart to minimise the potential for cognitive decline. MMSE groupings were defined using three different criteria and the study was population-based using data on 2,275 individuals from five sites across England and Wales.

## Methods

### Study population

Data came from the Medical Research Council Cognitive Function and Ageing Study (MRC CFAS) [12]. Briefly, MRC CFAS is a multi-centre study on over 18,000 persons from across six centres in England and Wales; five of the centres have the same standardised design. These centres used a two-phase sampling design with a screening interview followed by an assessment interview. Participants were selected from Family Health Service Authority lists and were stratified by age to include persons aged 65 years and over at the index date for each centre and living within a specified geographical area. The study began in the late 1980s; baseline interviews took place between 1989 and 1993.

In this study data were used from the five centres with a standardised design: Cambridgeshire, Gwynedd, Newcastle, Nottingham, and Oxford (total n = 13,004). The population under investigation contained individuals who were cognitively assessed at the baseline screening interview or the assessment interview around two months later (n = 2,640, both tests were completed by 2,275 participants). The population invited to the assessment interview was weighted towards those in a potentially frail cognitive state (identified using details from the screen interview, including MMSE scores) although all levels of ability were represented. For full details of the questionnaires used at the screen and assessment waves please see http://www.cfas.ac.uk.

### Cognitive Assessment

The Mini-Mental State Examination (MMSE) [1] was administered to participants at both the screen and assessment interviews. The version of the MMSE used in this study included serial sevens, but not spelling 'world' backwards [13]. The words to repeat and recall were 'apple, penny, table' at screen, and 'tree, clock, boat' at assessment. Items that could not be answered due to sensory or mobility problems were considered failed, all other items that were not answered were kept as missing data [12]. Incomplete MMSE scores tend to come from individuals who are severely cognitively impaired.

MMSE scores range from 0-30 and there have been several definitions proposed to categorise these scores into cognitive states. The three definitions used in this paper were suggested by *MRC CFAS, Tombaugh and McIntyre *[5] and *Folstein et al*. [13]. The *MRC CFAS *categorisation was based on the ROC curve findings from Figure One of Stephan et al. 2010 [14], which showed the MMSE to be as accurate as other diagnostic definitions of Mild Cognitive Impairment in predicting future risk of dementia. The graph indicated MMSE groupings as follows: < 18 (severe impairment), 18-22 (moderate impairment), 23-26 (slight impairment), 27-30 (no impairment). *Folstein et al*. who devised the MMSE [1] also recommended splitting the MMSE scores into four groups (< 11 severe impairment, 11-20 moderate impairment, 21-26 mild impairment, 27-30 no impairment) while *Tombaugh and McIntyre's *seminal review reported a trend towards a three group categorisation (< 18 severe impairment, 18-23 mild impairment, 24-30 no impairment).

### Interview Administration

Interviewers at both screen and assessment had a range of backgrounds, mainly professions allied to medicine. These included psychologists, psychiatrists, registered nurses and others with similar backgrounds. All interviewers received identical training from the CFAS study co-ordinators. Wording, prompting and feedback were all strictly controlled by a combination of training and computer assisted interviewing. Monitoring of the quality and consistency of interviews was carried out to ensure comparability both within and between centres through observation, role play, and analysis of audiotapes of interviews in the field. Interviews took place in the respondents' homes.

### Statistical Methods

MMSE scores were categorised into groups, which were relabelled in ascending order from 1 (low cognition) to 4 (high cognition) (or 1 to 3). Cognitive change was measured by subtracting the assessment group number from the screen group number. This created a scoring range of -3 to 3 (or -2 to 2) where 0 represented no change in group. Descriptive statistics were used to compare the classification performance of each categorisation method.

To determine whether baseline cognitive score had an effect on cognitive change, weighted logistic regression was used to test for differences between those who changed group compared to those who did not. Age, sex, and study centre were entered as covariates along with the MMSE score from the screen interview and the duration in months between screen and assessment interviews. Inverse probability weights were calculated using logistic regression-study participation was regressed on age, sex, screening MMSE score, and GMS-AGECAT (Geriatric Mental State-Automated Geriatric Examination for Computer Assited Taxonomy), which is a computerised diagnostic system that can be used to diagnose dementia. This enabled the cohort under investigation to be back-weighted to the original population-based cohort of 13,004 individuals. All analyses were conducted in R version 2.10.1 [15].

## Results

The characteristics of the population are presented in Table 1. There were more women than men in the study population (63%, n = 1,442) and more people in the younger age-groups (23%, n = 514 in the 65-69 year old group compared to 5%, n = 103 in the > 90 years old group). The time between MMSE assessments ranged from 5 to 630 days; the median time was 69 days, inter-quartile range 45 to 111. Finally, the median score at the screen MMSE was 24 (inter-quartile range 20 to 25) although scoring covered the entire MMSE range (0-30). The maximum difference between screen and assessment scores was twelve points (median change 0, IQR -2 to 2). The weighted Pearson correlation between the screen and assessment MMSE scores was 0.79 (0.76 0.82); Spearman's rank correlation was 0.74. Cronbach's α measure of reliability was 0.91 (0.90, 0.92).

**Table 1:** Characteristics of the CFAS analysis population with valid MMSE scores at baseline screen and assessment

	Total sample (n = 13,004)		Analysis sample (n = 2,275)	
	n	%	n	weighted %
Age group (years)				
65-69	3,184	24	514	24
70-74	3,150	24	559	24
75-79	2,906	22	425	23
80-84	2,256	17	415	17
85-89	1,092	8	259	9
≥ 90	416	3	103	4
Sex-men	5,157	40	833	39
Education < 9 years	872	7	209	7
Social class grouping-manual	7,152	56	1,435	59
Baseline/screen MMSE < 21	1,180	9	587	11
Days between screen and assessment*	-	-	69	(45, 111)

*median (quartiles)

### Tombaugh and McIntyre categorisation

The number (and weighted percentage) of participants in each cognitive category are shown in Table 2. Seventy-eight percent were classified in the same group at both time-points with 14% moving up a group and 8% moving down a group. The proportion of people moving up or down more than one group was negligible. For individuals who did not change cognitive group, 90% scored within three points of their initial MMSE at follow-up; just under half (48%) of those who moved up or down one group scored within three points of their initial mark (results not shown).

**Table 2:** Classification of MMSE states at screen and assessment waves*

Tombaugh and McIntyre criteria									
		MMSE at assessment							
		< 18		18-23		24-30			
MMSE at screen	< 18	237	(3%)	56	(3%)	2	(< 0.1%)		
	18-23	132	(1%)	451	(13%)	228	(11%)		
	24-30	6	(< 0.1%)	208	(7%)	955	(62%)		
									
**Folstein et al. criteria**									
		MMSE at assessment							
		< 10		11-20		21-26		27-30	

MMSE at screen	< 10	39	(< 1%)	13	(1%)	0		0	
	11-20	37	(< 1%)	373	(7%)	122	(6%)	3	(1%)
	21-26	0		165	(3%)	763	(31%)	289	(12%)
	27-30	0		6	(< 0.1%)	145	(13%)	320	(28%)
**MRC CFAS criteria**									
		MMSE at assessment							
		< 18		18-22		23-26		27-30	

MMSE at screen	< 18	237	(3%)	52	(3%)	6	(< 1%)	0	
	18-22	129	(1%)	305	(8%)	173	(7%)	18	(1%)
	23-26	8	(< 0.1%)	173	(5%)	429	(19%)	274	(11%)
	27-30	1	(< 0.1%)	16	(< 1%)	134	(12%)	320	(28%)

*Number observed (weighted percentage)

### Folstein categorisation

The proportion of participants classified in the same cognitive group was 66%. A similar proportion of people moved either up (19%) or down (16%) one group with very few moving two or more groups (1%). When comparing the actual changes in cognitive scores as opposed to the changes by group, 95% of people who stayed in the same group at assessment were within three points of their initial MMSE (results not shown). For those who moved up or down one cognitive group, 56% were within three points of their initial MMSE score.

### MRC CFAS categorisation

The distribution of change in cognitive category is shown in Table 2. The data were symmetrical about the participants who remained in the same cognitive group (58%). Approximately 40% of the sample went up (21%) or down (18%) one cognitive group whilst ~2% moved by more than one group. The distribution of actual difference in cognitive scores showed that the majority of people who stayed in the same group scored within three points of their initial MMSE score (98%, results not shown). For those who moved up or down one cognitive group, the majority were also within three points of their initial MMSE score (63%).

### Logistic regression output

The weighted logistic regression analyses yielded few consistently significant predictors of change in cognitive group (Table 3). There were some modest associations between increased age and greater odds of moving group although these were only statistically significant for the *Tombaugh and McIntyre *criteria. MMSE scores at the screen interview were associated with changing cognitive group for the *Tombaugh and McIntyre *and *MRC CFAS *classifications (odds ratios 0.86, 95% confidence interval (0.84, 0.88), p < 0.001; 0.95 (0.93, 0.97), p < 0.001, respectively). There were no statistically significant associations between changing cognitive group and gender, or the time in months between screen and assessment interview. The regression output suggests a very slight centre effect for Nottingham. However, this is due to choosing the Cambridge centre as the reference group; a floating point analysis showed little difference between changing state and study centre (results not shown).

**Table 3:** Weighted logistic regression output for no change versus change in cognitive group

		Weighted Logistic Regression Odds Ratios and 95% Confidence Intervals		
		*Tomabugh and McIntyre*	***Folstein et al***.	*MRC CFAS*
Age group	65-69	1.0 (referrant)	-	-
	70-74	1.37 (1.01, 1.85) *	1.16 (0.87, 1.54)	1.17 (0.89, 1.53)
	75-79	1.81 (1.32, 2.49)^†^	1.22 (0.89, 1.67)	1.30 (0.96, 1.74)
	80-84	1.55 (1.08, 2.23) *	1.34 (0.96, 1.86)	1.21 (0.88, 1.67)
	85-89	1.72 (1.11, 2.67) *	1.12 (0.74, 1.69)	1.12 (0.76, 1.65)
	≥ 90	0.85 (0.41, 1.72)	0.97 (0.53, 1.77)	0.56 (0.31, 1.02)
Sex	Men	1.01 (0.81, 1.26)	0.91 (0.74, 1.13)	0.93 (0.76, 1.13)
Centre	Cambridge	1.0 (referrant)	-	-
	Gwynedd	1.08 (0.77, 1.51)	1.30 (0.94, 1.79)	1.28 (0.94, 1.75)
	Newcastle	0.75 (0.53, 1.06)	0.91 (0.65, 1.28)	0.96 (0.70, 1.32)
	Nottingham	1.16 (0.82, 1.64)	1.43 (1.01, 2.00) *	1.39 (1.01, 1.93) *
	Oxford	0.89 (0.63, 1.27)	1.08 (0.76, 1.52)	1.10 (0.79, 1.54)
Screen MMSE score		0.86 (0.84, 0.88)^†^	0.99 (0.97, 1.02)	0.95 (0.93, 0.97)^†^
Months between screen and assessment		1.05 (0.99, 1.11)	1.00 (0.95, 1.05)	1.00 (0.95, 1.05)

*P < 0.05, ^† ^P < 0.001

## Discussion

This study investigated the reliability of the Mini-Mental State Examination (MMSE) using three state-based categorisations on 2,275 older persons from a population-based study from five sites across England and Wales. The number of individuals classified in the same state two months after an initial screen assessment varied from 57% (*MRC CFAS*), to 65% (*Folstein et al*.), to 78% (*Tombaugh and McIntyre*). The proportions of participants who went either up or down a single group were similar with a minimal number moving up or down more than one group. The reliability of state-based groupings is moderate-to-good and similar to statistics obtained from correlation or Cronbach-α analyses.

There was no significant predictor of changing group across all three models although higher original MMSE scores were associated with reduced change in the *MRC CFAS *and *Tombaugh and McIntyre *classifications. This inverse association in the former case was very weak whilst in the latter case it is most likely due to the large range of values lying within their non-impaired state (MMSE score between 24 and 30).

The greatest reliability was found using the *Tombaugh *criteria although this had much to do with their classification method using three cognitive groups as opposed to four. Indeed, there is very little difference between the four-state approaches. The slightly poorer performance of the *MRC CFAS *classification is most likely due to the use of smaller bands for the cognitive groupings at the higher level of scoring-where most of the data points lie in the general population. This again implies that most of the change occurs around the cut-points-an issue raised by Van Den Hout and Matthews who split cognition into two groups based around a cut-point between 21 and 22 for a two-state illness-death multi-state model [16].

It is common for MMSE scores less than 18 to be used as an indication of severe impairment in healthy populations. How the MMSE is categorised at its upper levels is more contentious, particularly with regards to attempts to identify individuals with MCI. Recent studies have shown there to be many different definitions of MCI [17] with progression rates to dementia dependent on which scale has been used [18]. It has been shown that an MMSE group between 23 and 26 performs as well as other, more complex methods of MCI classification in prediction of future dementia [14]. This justifies its place as a valuable tool in the assessment of cognitive ability and highlights the importance of understanding its reliability. It also highlights the usefulness of the *MRC CFAS *criteria applied in this paper where one of the groups contained MMSE scores between 23 and 26.

The strengths of the investigation include the application of two commonly applied MMSE categorisation models along with the *MRC CFAS *groupings to a large population-based sample of older persons. In addition to this being the first time that state-based variation of the MMSE has been investigated over a short follow-up period, we also looked at actual variation about scores-most were found to lie within three points of each other. A previous analysis that examined differences by MMSE groupings found a regression to the mean effect [19]. However, the elapsed time between interviews was five years-a period too long to assess test-retest reliability in older people as actual cognitive change is likely to have occurred during this time.

A potential limitation of the study was the duration of time between the cognitive measures and the age of participants in the study. However, the former was not significant in any of the logistic regression models that attempted to identify those who changed group, and in addition a sensitivity analysis using a cut-point of 60 days between screen and assessment showed the same effects. There was some inconsistent evidence of an association between changing group and age; with younger people were more likely to move group. This may have an impact upon the frequency of testing required to identify an 'at risk' population of younger participants. A limitation of using MMSE groups for analysing cognitive change in population-based studies is that the MMSE ceiling effect makes it difficult to assess successful cognitive ageing. However, this problem is also present in non state-based MMSE models. Finally, reliable change indices (RCIs) can also be used to assess cognitive change over time whilst adjusting for measurement error, practice effects, and regression to the mean [19,20]. However, the current analysis is motivated by the assignment of individuals to cognitive groups based on a single MMSE score. Future analyses will use the *MRC CFAS *state classification to assess longitudinal decline in abilities.

## Conclusions

Compared to correlation and Cronbach α statistics, a state-based approach to analysing the MMSE provides similar estimates of its reliability. However, the large proportion of participants with test re-test scores within three points of each other suggests that a state-based approach to modelling cognitive change using MMSE scores may help avoid bias in the form of regression to the mean. State-based models are therefore an ideal analysis tool when assessing longitudinal cognitive change using the MMSE.

## Competing interests

The authors declare that they have no competing interests.

## Authors' contributions

REM analysed and interpreted data, drafted the first version of the manuscript and revised and edited the manuscript. REM is guarantor of the analysis. MC contributed to design and revised and edited the manuscript. FEM contributed to design, advised on the analytical strategy, data acquisition, assisted obtaining funding and revised and edited the manuscript. CB conceived the research idea, contributed to design, data acquisition, funding and revised and edited the manuscript. All authors read and approved the final manuscript.

## Pre-publication history

The pre-publication history for this paper can be accessed here:

http://www.biomedcentral.com/1471-2288/11/127/prepub
